# Prevalence of non-communicable disease and the associated factors among healthcare workers in Qatar

**DOI:** 10.1186/s12875-025-02760-x

**Published:** 2025-03-10

**Authors:** Ayman Al-Dahshan, Noora Alkaabi, Sarah Naja, Nada Adil, Tharaa Al-Shammari, Haya Alkaabi

**Affiliations:** Preventive Medicine Division, Department of Medicine, Hamad Medical Corporation, Doha, Qatar

**Keywords:** Non-communicable diseases, Healthcare workers, Occupational health, Prevalence, Qatar

## Abstract

**Background:**

Non-communicable diseases (NCDs) significantly impact global health and contribute to economic burdens and premature deaths, with healthcare workers (HCWs) being at high risk. This study aims to assess the prevalence and associated factors of NCDs among newly hired HCWs at Hamad Medical Corporation (HMC) in Qatar.

**Methods:**

This retrospective cross-sectional study analyzed 3097 electronic medical records of newly hired HCWs at HMC during 2021 and 2022. Diagnoses were coded using ICD-10 and SNOMED. Descriptive statistics and Chi-square tests were used, with significance set at *p* < 0.05.

**Results:**

The mean age of participants was 31.8 years (SD ± 6.9), with almost equal numbers of males (50.1%) and females (49.9%). Indians made up 36.2%, and 63.9% were married. Professionally, 38% were nurses, 18.8% were physicians, and 11.2% were laboratory professionals. Overall, about one-third (30.9%) of the HCWs had at least one NCD. Diabetes mellitus (11.3%), thyroid disease (9.8%), and hypertension (7.4%) were the most frequent NCDs. Older individuals (≥ 40 years old) have higher comorbidity rates (47%) than younger groups (24%, *p* < 0.001). Females have higher rates (39.5%) compared to males (22.4%, *p* < 0.001). Married individuals, nationality, and occupation also significantly influence comorbidity, with administrative staff showing the highest prevalence of NCDs (45.5%, *p* = 0.011).

**Conclusions:**

Over 30% of the newly hired HCWs had an NCD, with diabetes, thyroid disease, and hypertension being most common. Higher prevalence was observed among older staff, females, married individuals, and administrative workers. Targeted workplace health programs are needed for early detection and prevention.

## Introduction

The priority of non-communicable diseases (NCDs) in worldwide policy has increased significantly. They pose a significant obstacle to achieving the Sustainable Development Goals (SDG), particularly target 3.4, which calls for reducing premature deaths from NCDs by one-third by 2030 through prevention and treatment [1]. NCDs are globally recognized as a threat to human well-being and quality of life, contributing to 74% of the global mortality rate and affecting 41 million people annually [2].

The World Health Organization (WHO) has adopted the “4 × 4 frame,” focusing on four common modifiable risk factors: harmful alcohol use, unhealthy eating, tobacco use, and physical inactivity. These factors are linked to the most common NCDs, namely cardiovascular diseases, cancers, chronic respiratory diseases, and diabetes [3]. The International Labor Organization emphasizes the workplace as a critical platform for health promotion and NCDs prevention [4].

In the Gulf Cooperation Council (GCC) countries, four major NCDs cause nearly 40,000 deaths annually, accounting for over 43% of all premature deaths [5]. The economic burden of NCDs among healthcare workers (HCWs) in the GCC is substantial, with direct costs of main NCDs estimated at $16.7 billion in 2019, and indirect costs from worker productivity losses reaching around $80 billion [6]. In Qatar, NCDs claimed 1,600 lives in 2018. The economic burden of NCDs in 2019 was QAR 18.1 billion (≈5 billion USD), which corresponds to 2.7% of the GDP. Healthcare expenditures for NCDs totaled QAR 7.2 billion, distributed among cardiovascular diseases (47%), diabetes (30%), chronic respiratory diseases (16%), and cancer (7%). Absenteeism due to NCDs cost an additional QAR 1.4 billion [7].

Chronic illness-related absences among HCWs can significantly impact the resilience of healthcare systems, potentially delaying the achievement of global SDGs due to the direct impact on public health [1, 8]. Healthcare providers serve as role models for healthy lifestyles and are vital to creating a better society [9]. HCWs face high risks of NCDs due to reasons such as prolonged setting, mental stress and physical exhaustion from overtime and work shifts [10].

Occupation is a significant risk factor for NCDs. For example, a study in Taiwan found that medical professionals had a significantly higher prevalence of hypertension, with an odds ratio of 1.74 (95% CI = 1.05–2.91) compared to non-medical workers [11]. Additionally, myocardial infarction and angina are more likely in obese male physicians/surgeons in managerial positions and female managers [12]. Most work-related diseases are non-communicable, with cardiovascular diseases (31%), cancers (26%), and respiratory diseases (17%) accounting for nearly three-quarters of work-related mortality worldwide [13]. A multi-country cross-sectional study in sub-Saharan Africa found that the prevalence of at least one chronic disease among HCWs ranged from 9.7% in Nigeria to 20.6% in Madagascar [14].

In Qatar, a study conducted by Hamad Medical Corporation (HMC), the country’s primary public healthcare provider, during the COVID-19 pandemic revealed that 69.7% of female and 70.9% of male HCWs were overweight, and 55.2% did not engage in any physical activity [15]. Previous research has primarily focused on lifestyle behaviors, leaving a gap in understanding the burden of NCDs within the healthcare workforce. The WHO and Qatar National Strategy (2018–2022) emphasize the workplace as a priority setting for health promotion and protection, aligning with national goals [16, 17]. Given the lack of comprehensive data on NCDs among HCWs, this study amis to assess the prevalence of NCDs and the associated factors among newly hired HCWs who attended the pre-employment clinic at Hamad Medical Corporation.

## Methodology

### Study design and sample size

The study utilized a retrospective cross-sectional design. The study included all new staff members who visited the pre-employment clinic at the Staff Medical Center (SMC) within HMC from January 1, 2021, to November 15, 2022. No formal sample size calculation was performed. Instead, we analyzed all available electronic medical records (EMRs) of individuals who underwent pre-employment health assessments during this period. Additionally, no exclusion criteria were applied, as all eligible records were included in the analysis.

### Data source

The primary data source for this study comprised EMRs from pre-employment encounters of staff members at the SMC. These medical records contain comprehensive information about the health assessments conducted during pre-employment visits, including medical history, physical examinations, laboratory test results, and other health screenings. The utilization of EMRs ensured standardized documentation and facilitated efficient data retrieval for analysis. Additionally, the electronic format allowed for systematic data management and quality control measures.

### Study population and study setting

The study population included all new staff who visited the pre-employment clinic in the SMC within HMC during the study period. HMC is the main provider of secondary and tertiary healthcare in Qatar, managing a comprehensive network of 12 hospitals [18]. The SMC is dedicated to providing comprehensive medical services to HMC employees, including general medical consultations, preventive medicine, health screenings, and occupational medicine services. A key feature of the center is its provision of pre-employment health assessments for all HMC staff, ensuring that new employees meet necessary health standards before starting their roles.

### Study procedures

The de-identified data were retrieved from the EMRs with the support of the Business Intelligence Unit at HMC. To ensure data accuracy and integrity, a multi-step data management process was implemented. This included identifying and resolving any discrepancies or missing information, verifying the consistency of data, and conducting quality control checks. The data were then entered by the research team into a pre-designed data collection sheet for cleaning, verification, categorization of variables and coding of diagnoses. After ensuring data integrity, the finalized dataset was entered into SPSS (Statistical Package for the Social Sciences) for statistical analysis.

### Measurement

#### Dependent variables

Diagnoses of NCDs in this study were identified based on documented medical histories in the EMRs and were coded using the International Classification of Diseases, 10th Revision (ICD-10), and the Systematized Nomenclature of Medicine (SNOMED). These comorbidities included prediabetes, diabetes, hypertension, cardiovascular diseases, dyslipidemia, cancer, thyroid disease, chronic lung diseases, chronic kidney disease and mental health conditions (depression, anxiety, stress).

#### Independent variables

The independent variables included sociodemographic characteristics obtained from the EMRs. These variables included *age* (recorded in years), *sex* (male or female), *nationality* (classified by country of origin), *marital status* (single, married, or divorced), *job title* (position within HMC), and the *appointment date* (the date when the staff member joined HMC).

### Ethical considerations

The study obtained ethical clearance from the Institutional Review Board of Hamad Medical Corporation [Reference number: MRC-01–23-007]. All collected data were anonymized. The research was conducted in accordance with the Declaration of Helsinki. The collected patient data was kept confidential and used solely for the intended purposes, in compliance with patient privacy laws of HMC and the Ministry of Public Health (MoPH), Qatar. To protect participant confidentiality and prevent potential misuse of their information, data provided for this study was de-identified and remained so even after the study was completed.

### Statistical consideration and data analysis

For statistical analysis, the finalized de-identified dataset was imported into SPSS. Both descriptive and analytic statistics was applied. For the descriptive statistics, summarization will utilize frequency distribution tables, percentages, mean, and Standard Deviation (SD) whenever appropriate. For analytical statistics, Chi-square test (χ^2^) was applied for categorical data. Statistical significance will be considered at *p* < 0.05 as a cutoff point.

## Results

Table 1 presents the background characteristics of new staff attending the SMC at HMC (*N* = 3,097). The mean age of these participants was 31.8 years (SD ± 6.9), with an almost equal proportion of males (50.1%) and females (49.9%). A significant proportion of the new staff were Indian, making up about 36.2% (*n* = 1,122) of the participants, and the majority were married (63.9%; *n* = 1,979). Regarding professional roles, about 38% (*n* = 1,185) of the new staff were nurses, followed by physicians at 18.8% (*n* = 582) and laboratory professionals at 11.2% (*n* = 349), as detailed in Table 1.

**Table 1:** Background characteristics and distribution of comorbidities among new staff attending staff medical center at Hamad Medical Corporation (*N* = 3,097)

Variable	Frequency	Percent
**Age (years)**		
< 30	1,320	42.7
30–39	1,422	46.0
≥ 40	351	11.3
*mean* ± *SD*	*31.8* ± *6.9*	
**Sex**		
Female	1,546	49.9
Male	1,551	50.1
**Marital status**		
Single	1,096	35.4
Married	1,979	63.9
Divorced	22	0.7
**Nationality**		
Indian	1,122	36.2
Filipino	535	17.3
Jordanian	288	9.3
Sudanese	217	7.0
Qatari	142	4.6
Egyptian	73	2.4
Pakistani	82	2.6
Tunisian	81	2.6
Egyptian	73	2.4
Syrian	65	2.1
Lebanese	55	1.8
Bangladeshi	30	1.0
Others	334	10.8
**Occupation**		
Nurse	1,185	38.3
Physician	582	18.8
Technician professional	349	11.2
Paramedic	289	9.3
Patient care attendant	249	8.0
Administrative	101	3.3
Pharmacist	92	3.0
Physiotherapist	58	1.9
Occupational therapist	58	1.9
Researcher/analyst	37	1.2
Respiratory therapist	31	1.0
Others^a^	66	2.1
**Presence of any comorbidities**		
Yes	957	30.9
No	2,140	69.1
**Number of comorbidities (*n* = 957)**		
One comorbidity	643	67.2
Two comorbidities	219	22.1
Three or more comorbidities	95	9.2
**Diabetes mellitus**	349	11.3
**Thyroid disease**	302	9.8
**Hypertension**	228	7.4
**Prediabetes**	130	4.2
**Dyslipidemia**	116	3.7
**Chronic lung disease**^**b**^	101	3.3
**Anxiety**	65	2.1
**Depression**	32	1.0
**Cardiovascular disease**	18	0.6
**Chronic kidney disease**	14	0.5
**Cancer**	13	0.4
**Stress**	12	0.4

^a^Include: dental assistant, dietitian, speech therapist, scientist

^b^Includes chronic lung diseases other than asthma: COPD, interstitial lung disease, and bronchiectasis

Regarding health-related characteristics, it is noteworthy that approximately one-third of the new staff 31% (*n* = 957) had pre-existing chronic health conditions. Among these, diabetes mellitus was the most frequently chronic illness, affecting 11.3% of the new staff, followed by thyroid disease (9.8%) and hypertension (7.4%), as detailed in Table 1.

Table 2 shows the prevalence of NCDs by sociodemographic and occupational characteristics of new staff. Older individuals (40 years and above) have a higher comorbidity rate (47%) than middle-aged (33.3%) and younger (24%) groups (*p* < 0.001). Females show higher comorbidity (39.5%) compared to males (22.4%), with a *p*-value < 0.001. Marital status and nationality significantly influence comorbidity rates. Administrative staff have the highest prevalence of comorbidities (45.5%), while paramedics (23.9%) and therapists (26.4%) have lower rates (*p* = 0.011).

**Table 2:** Prevalence of comorbidities according to sociodemographic and occupation characteristics

**Variable**	**Has NO comorbidity,** n (%)	**Has ANY comorbidity, n** (%)	***p*-value**
**Age (years)**			< 0.001*
< 30	1003 (76.0)	317 (24.0)	
30–39	948 (66.7)	474 (33.3)	
≥ 40	186 (53.0)	165 (47)	
**Sex**			< 0.001*
Male	1204 (77.6)	347 (22.4)	
Female	936 (60.5)	610 (39.5)	
**Marital status**			< 0.001*
Married	1282 (64.8)	697 (35.2)	
Unmarried	858 (76.8)	259 (23.2)	
**Nationality**			< 0.001*
Indian	746 (66.5)	375 (33.5)	
Filipino	381 (71.2)	154 (28.8)	
Jordanian	222 (77.1)	66 (22.9)	
Sudanese	153 (70.5)	64 (29.5)	
Qatari	69 (48.6)	73 (51.4)	
Egyptian	37 (50.7)	36 (49.3)	
Pakistani	54 (65.9)	28 (34.1)	
Tunisian	65 (80.2)	16 (19.8)	
Syrian	65 (80.2)	17 (26.2)	
Lebanese	50 (90)	5 (9.1)	
Bangladeshi	24 (80)	6 (20)	
Others	210 (73.2)	77 (26.8)	
**Occupation**			0.011*
Nurse	802 (67.7)	383 (32.3)	
Physician	403 (69.2)	179 (30.8)	
Technician professional	236 (67.6)	113 (32.4)	
Paramedic	220 (76.1)	69 (23.9)	
Patient care attendant	194 (70)	83 (30)	
Therapist	117 (73.6)	42 (26.4)	
Administrative staff	55 (54.5)	46 (45.5)	
Pharmacist	69 (75)	23 (25)	
Researcher	33 (70.2)	14 (29.8)	
Dietician	11 (68.8)	5 (31.3)	

^*^statistically significance

Table 3 presents the association between the history of chronic diseases and the age of new staff, highlighting several statistically significant findings related to older age. For instance, hypertension was significantly higher among participants aged ≥ 40 years compared to the younger age groups (25.4% vs. 7.3% vs. 2.7%, *p*-value < 0.001). Similarly, diabetes was more prevalent among participants aged ≥ 40 years (19.7%) compared to those aged 30–39 years (13.3%) and those aged up to 29 years (6.6%), with the difference being statistically significant (*p*-value < 0.001). Dyslipidemia was also significantly higher in the older age group (11.7%) compared to staff aged 30–39 years (3.8%) and those aged 29 years and younger (1.6%). Also, cardiovascular diseases were more frequently observed among staff aged 40 years and older (2.0%) compared to those aged 30–39 years (0.6%) and those aged 29 years and younger (0.2%), with a *p*-value of 0.001.

**Table 3:** The association between chronic diseases and the age of the new staff attending staff medical center at Hamad Medical Corporation (*N* = 3,097)

**Variable**	** ≤ 29,** n (%)	**30–39,** n (%)	** ≥ 40,** n (%)	***P*-value**
**Any chronic disease**				< 0.001*
Yes	317 (24.0%)	474 (33.3%)	165 (47.0%)	
No	1003 (76.0%)	948 (66.7%)	186 (53.0%)	
**Hypertension**				< 0.001*
Yes	35 (2.7%)	104 (7.3%)	89 (25.4%)	
No	1285 (97.3%)	1318 (92.7%)	262 (74.6%)	
**Pre-Diabetes**				< 0.001*
Yes	26 (2.0%)	76 (5.3%)	27 (7.7%)	
No	1294 (98.0%)	1346 (94.7%)	324 (92.3%)	
**Diabetes**				< 0.001*
Yes	87 (6.6%)	192 (13.5%)	69 (19.7%)	
No	1233 (93.4%)	1230 (86.5%)	282 (80.3%)	
**Dyslipidaemia**				< 0.001*
Yes	21 (1.6%)	54 (3.8%)	41 (11.7%)	
No	1299 (98.4%)	1368 (96.2%)	310 (88.3%)	
**CVD**				0.001*
Yes	3 (0.2%)	8 (0.6%)	7 (2.0%)	
No	1317 (99.8%)	1414 (99.4%)	344 (98.0%)	
**Chronic lung disease**				0.001*
Yes	61 (4.6%)	33 (2.3%)	7 (2.0%)	
No	1259 (95.4%)	1389 (97.7%)	344 (98.0%)	
**Chronic kidney disease**				0.374
Yes	4 (0.3%)	7 (0.5%)	3 (0.9%)	
No	1316 (99.7%)	1415 (99.5%)	348 (99.1%)	
**Thyroid disease**				0.057*
Yes	109 (8.3%)	153 (10.8%)	39 (11.1%)	
No	1211 (91.7%)	1269 (89.2%)	312 (88.9%)	
**Stroke**				0.617
Yes	1 (0.1%)	2 (0.1%)	1 (0.3%)	
No	1319 (99.9%)	1420 (99.9%)	350 (99.7%)	
**Cancer**				0.003*
Yes	0 (0.0%)	9 (0.6%)	4 (1.1%)	
No	1320 (100.0%)	1413 (99.4%)	347 (98.9%)	
**Depression**				0.172
Yes	18 (1.4%)	13 (0.9%)	1 (0.3%)	
No	1302 (98.6%)	1409 (99.1%)	350 (99.7%)	
**Anxiety**				< 0.001*
Yes	43 (3.3%)	19 (1.3%)	3 (0.9%)	
No	1277 (96.7%)	1403 (98.7%)	348 (99.1%)	
**Stress**				0.026*
Yes	8 (0.6%)	1 (0.1%)	12 (0.4%)	
No	1312 (99.4%)	1421 (99.9%)	348 (99.1%)	

^*^statistically significance

Other chronic disease were also associated with the age group as illustrated in Table 3.

Table 4 presents the association between the chronic diseases and the sex. Overall, chronic diseases were higher among female (39.5%) staff compared to male staff (22.4%) with this observation being statistically significant (*p*-value = 0.005).

**Table 4:** The association between chronic diseases and the sex of the new staff attending staff medical center at Hamad Medical Corporation (*N* = 3,097)

**Variable**	**Male,** n (%)	**Female,** n (%)	***P*-value**
**Any chronic diseases**			< 0.001*
Yes	347 (22.4%)	610 (39.5%)	
No	1204 (77.6%)	936 (60.5%)	
**Hypertension**			< 0.001*
Yes	138 (8.9%)	90 (5.8%)	
No	1413 (91.1%)	1456 (94.2%)	
**Pre-Diabetes**			0.843
Yes	64 (4.1%)	66 (4.3%)	
No	1487 (95.9%)	1480 (95.7%)	
**Diabetes**			< 0.001*
Yes	90 (5.8%)	259 (16.8%)	
No	1461 (94.2%)	1287 (83.2%)	
**Dyslipidaemia**			0.005*
Yes	73 (4.7%)	43 (2.8%)	
No	1478 (95.3%)	1503 (97.2%)	
**CVD**			0.158
Yes	12 (0.8%)	6 (0.4%)	
No	1539 (99.2%)	1540 (99.6%)	
**Chronic lung disease**			< 0.001*
Yes	23 (1.5%)	78 (5.0%)	
No	1528 (98.5%)	1468 (95.0%)	
**Chronic kidney disease**			0.008*
Yes	12 (0.8%)	2 (0.1%)	
No	1539 (99.2%)	1544 (99.9%)	
**Thyroid disease**			< 0.001*
Yes	51 (3.3%)	251 (16.2%)	
No	1500 (96.7%)	1295 (83.8%)	
**Stroke**			0.997
Yes	2 (0.1%)	2 (0.1%)	
No	1549 (99.9%)	1544 (99.9%)	
**Cancer**			0.163
Yes	4 (0.3%)	9 (0.6%)	
No	1547 (99.7%)	1537 (99.4%)	
**Depression**			< 0.001*
Yes	5 (0.3%)	27 (1.7%)	
No	1546 (99.7%)	1519 (98.3%)	
**Anxiety**			0.001*
Yes	19 (1.2%)	46 (3.0%)	
No	1532 (98.8%)	1500 (97.0%)	
**Stress**			0.020*
Yes	2 (0.1%)	10 (0.6%)	
No	1549 (99.9%)	1536 (99.4%)	

*statistically significance

Diabetes was reported at significantly higher percentages among female staff compared to male staff (16.8% vs. 5.8%, *p*-value < 0.001). Similarly, thyroid disease was significantly more frequent among female staff (16.2%) compared to males (3.3%). Chronic lung diseases were also more frequently observed among female staff compared to males (5.0% vs. 1.5%, *p*-value < 0.001).

Conversely, hypertension was significantly higher among male participants than female staff (8.9% vs. 5.8%, *p*-value < 0.001). Dyslipidemia was also more prevalent among male participants (4.7%) compared to female staff (2.8%), with the difference being statistically significant (*p*-value = 0.005).

Table 5 illustrates the association between health-related characteristics and the professions of new staff, revealing significant disparities in the prevalence of prediabetes, diabetes, chronic lung diseases, and thyroid diseases across different occupations.

**Table 5:** The association between history of chronic diseases and staff occupation attending staff medical center at Hamad Medical Corporation (*N* = 3,097)

	**Occupation, n (%)**									
**Chronic Disease**	**Nurse**	**Physician**	**Technician**	**Paramedic**	**Pharmacist**	**Dietitian**	**Therapist**	**Admin**	**Researcher**	***P*-value**
**Any chronic diseases**										0.011*
No	802 (67.7)	403 (69.2)	236 (67.6)	220 (76.1)	69 (75.0)	11 (68.8)	117 (73.6)	55 (54.5)	33 (70.2)	
Yes	383 (32.3)	179 (30.8)	113 (32.4)	69 (23.9)	23 (25.0)	5 (31.3)	42 (26.4)	46 (45.5)	14 (29.8)	
**Hypertension**										0.695
No	1108 (93. 5)	531(91.2)	322 (92.3)	265 (91.7)	85 (92.4)	16 (100.0)	150 (94.3)	93 (92.1)	45 (95.7)	
Yes	77 (6.5)	51 (8.8)	27 (7.7)	24 (8.3)	7 (7.6)	0 (0.0)	9 (5.7)	8 (7.9)	2 (4.3)	
**Prediabetes**										0.026*
No	1138 (96. 0)	564 (96.6)	332 (95.1)	278 (96.2)	91 (98.9)	16 (100.0)	155 (97.5)	92 (91.1)	44 (93.6)	
Yes	47 (4.0)	18 (3.1)	17 (4.9)	11 (3.8)	1 (1.1)	0 (0.0)	4 (2.5)	9 (8.9)	3 (6.4)	
**Diabetes**										< 0.001*
No	1033 (87. 2)	529 (90.9)	305 (87.4)	275 (95.2)	88 (95.7)	16 (100.0)	142 (89.3)	80 (79.2)	41 (87.2)	
Yes	152 (12.8)	53 (9.1)	44 (12.6)	14 (4.8)	4 (4.3)	0 (0.0)	17 (10.7)	21 (20.8)	6 (12.8)	
**Dyslipidaemia**										0.237
No	1154 (97. 4)	552 (94.8)	338 (96.8)	277 (95.8)	89 (96.7)	15 (93.8)	150 (94.3)	98 (97.0)	44 (93.6)	
Yes	31 (2.6)	30 (5.2)	11 (3.2)	12 (4.2)	3 (3.3)	1 (6.3)	9 (5.7)	3 (3.0)	3 (6.4)	
**CVD**										0.459
No	1181 (99. 7)	576 (99.0)	348 (99.7)	285 (98.6)	92 (100.0)	16 (100.0)	158 (99.4)	101 (100. 0)	47 (100.0)	
Yes	4 (0.3)	6 (1.0)	1 (0.3)	4 (1.4)	0 (0.0)	0 (0.0)	1 (0.6)	0 (0.0)	0 (0.0)	
**Chronic lung disease**										0.003*
No	1155 (97.5)	553 (95.0)	341 (97.7)	285 (98.6)	88 (95.7)	14 (87.5)	153 (96.2)	93 (92.1)	47 (100.0)	
Yes	30 (2.5)	29 (5.0)	8 (2.3)	4 (1.4)	4 (4.3)	2 (12.5)	6 (3.8)	8 (7.9)	0 (0.0)	
**Chronic kidney disease**										0.270
No	1181 (99.7)	581 (99.8)	346 (99.1)	285 (98.6)	91 (98.9)	16 (100.0)	159 (100.0)	101 (100. 0)	47 (100.0)	
Yes	4 (0.3)	1 (0.2)	3 (0.9)	4 (1.4)	1 (1.1)	0 (0.0)	0 (0.0)	0 (0.0)	0 (0.0)	
**Thyroid disease**										< 0.001*
No	1032 (87.1)	535 (91.9)	318 (91.1)	278 (96.2)	85 (92.4)	14 (87.5)	147 (92.5)	87 (86.1)	41 (87.2)	
Yes	153 (12.9)	47 (8.1)	31 (8.9)	11 (3.8)	7 (7.6)	2 (12.5)	12 (7.5)	14 (13.9)	6 (12.8)	
**Stroke**										0.723
No	1184 (99.9)	580 (99.7)	348 (99.7)	289 (100.0)	92 (100.0)	16 (100.0)	159 (100.0)	101 (100. 0)	47 (100.0)	
Yes	1 (0.1)	2 (0.3)	1 (0.3)	0 (0.0)	0 (0.0)	0 (0.0)	0 (0.0)	0 (0.0)	0 (0.0)	
**Cancer**										0.511
No	1180 (99.6)	579 (99.5)	348 (99.7)	289 (100.0)	92 (100.0)	16 (100.0)	158 (99.4)	99 (98.0)	47 (100.0)	
Yes	5 (0.4)	3 (0.5)	1 (0.3)	0 (0.0)	0 (0.0)	0 (0.0)	1 (0.6)	2 (2.0)	0 (0.0)	
**Depression**										0.199
No	1178 (99.4)	571 (98.1)	345 (98.9)	286 (99.0)	91 (98.9)	15 (93.8)	157 (98.7)	100 (99.0)	47 (100.0)	
Yes	7 (0.6)	11 (1.9)	4 (1.1)	3 (1.0)	1 (1.1)	1 (6.3)	2 (1.3)	1 (1.0)	0 (0.0)	
**Anxiety**										0.296
No	1167 (98.5)	564 (96.6)	338 (96.8)	282 (97.6)	90 (97.8)	15 (93.8)	156 (98.1)	99 (98.0)	46 (97.9)	
Yes	18 (1.5)	18 (3.1)	11 (3.2)	7 (2.4)	2 (2.2)	1 (6.3)	3 (1.9)	2 (2.0)	1 (2.1)	
**Stress**										0.753
No	1179 (99.5)	577 (99.1)	348 (99.7)	289 (100.0)	92 (100.0)	16 (100.0)	159 (100.0)	101 (100.0)	47 (100.0)	
Yes	6 (0.5)	5 (0.9)	1 (0.3)	0 (0.0)	0 (0.0)	0 (0.0)	0 0 (0.0)	0 (0.0)	0 (0.0)	

^*^statistically significance

Prediabetes was more prevalent among administrative staff (8.9%), and physician assistants (7.2%), than among other professionals, and this difference was statistically significant (*p*-value = 0.026). In addition, diabetes mellitus was significantly highly reported among administrative staff (20.8%), followed by physician assistants (13.7%), nurses (12.8%), researchers/analysts (12.8%), and technicians (12.6%), compared to other occupations, with a *p*-value of less than 0.001.

Regarding chronic lung diseases, the proportion of individuals with a history of such illnesses was higher among dietitians (12.5%), and among administrative staff (7.9%), compared to those in other occupations, with a *p*-value of 0.003.

Furthermore, thyroid disease was significantly more prevalent among administrative staff (13.9%), nurses (12.9%), researchers/analysts (12.8%), and dietitians (12.5%) compared to other occupations, with a *p*-value of less than 0.001, as shown in Table 5.

## Discussion

The present study assessed the prevalence of non-communicable diseases (NCDs) and associated factors among 3,097 healthcare workers attending pre-employment services at Hamad Medical Corporation (HMC) in Qatar. Approximately one-third of the new staff had at least one NCD. Older age (≥ 40 years), female gender, marital status, and certain occupations such as administrative, technician, and nursing roles were associated with a higher likelihood of having NCDs.

Qatar has witnessed an increasing demand for labor since hosting the 2022 FIFA World Cup. Similar to other Gulf Cooperation Council (GCC) countries, the estimated direct medical costs for seven major NCDs among employees amount to 0.6% of the gross domestic product, or $16.7 billion (2019 International $). As the burden of NCDs increases, so will the associated costs [19].

Workplace strategies are crucial in tackling NCDs. Plans for 'Healthy and Safe Employees' and providing safer workplaces are priorities in Qatar's National Health Strategy 2018–2022 [20, 21]. HMC, a leading not-for-profit healthcare provider in Qatar, operates 12 hospitals serving both nationals and expatriates [22]. The prevalence of NCDs among new healthcare staff attending the pre-employment clinic at HMC is almost 31%, which is triple the rate reported by the WHO STEP-wise survey among skilled employees (10.15%) [23].

Our data are comparable to the latest study among migrant blue-collar workers (31%) selected from six primary healthcare clinics in Qatar. This finding was unexpected, as it was anticipated that unskilled workers would be at higher risk for the four selected NCDs compared to skilled workers [24, 25]. Compared to regular patients attending primary healthcare centers, employees had double the burden of NCDs, with 16.2% of patients having one or more NCDs [26].

In the present study, diabetes was the most prevalent NCD, affecting about 11% of employees. This percentage is lower than the national prevalence of diabetes in Qatar, which was 17.8% in 2023 [27].

Significant differences in NCD prevalence between men and women were found. Women had higher prevalence rates of NCDs, including diabetes, thyroid disorders, chronic lung disease depression, and anxiety compared to men. Statistical significance was found in all NCDs (39.5% in women vs. 22.4% in men, *p* < 0.001). These findings suggest that NCDs are not primarily a disease of men, and workplace NCD screening programs should target women. This is especially relevant given that the absolute number of NCD deaths in women (16.2 million) is similar to that of men (18.4 million), as women tend to live longer by an average of 6 to 8 years [28].

About 2% of employees were affected by stress, depression, and anxiety. A previous study among healthcare workers in public health hospitals in Qatar found a higher prevalence of stress and depression, influenced by concerns about job descriptions, roles, and job security. The previous study's higher prevalence may also be due to the larger proportion of female participants (1260), as biological factors play a role [29].

Age was a crucial factor, with employees aged 40 and above having a significantly higher risk for NCDs compared to those below 40. This aligns with a meta-analysis showing that the prevalence of multi-morbidity increases with age [30]. Conversely, anxiety correlated negatively with age, possibly due to enhanced control of negative emotions in older adults, resulting in diminished limbic/emotional circuits and higher prefrontal cortical responses to negative emotions [31].

In our analysis, a chi-squared test (Table 2) indicated a significant association between nationality and NCDs. Higher proportions of NCD cases were observed among certain nationalities, including Qatari, Egyptian, Pakistani, Indian, and Filipino. However, nationality-specific prevalence rates were not calculated or directly compared. It is important to consider that the incidence rates of particular conditions in individuals' home countries may influence the current health outcomes of newly employed healthcare workers in Qatar. Ethnic and national backgrounds may contribute to variations in NCD prevalence, reflecting differing health profiles and environmental factors from their countries of origin [32], consistent with findings of previous research assessing NCD prevalence among adults visiting public primary healthcare centers in Qatar [26]. Regarding occupation, NCD prevalence was highest among administrative staff (44.5%) compared to other occupations, likely due to sedentary lifestyles and prolonged sitting, as observed in similar studies conducted in India [33]. Conversely, Paramedics exhibited the lowest rate of NCDs, aligning with previous findings that underscore the physical demands and active nature of their work [3, 4].

This research is the first study in Qatar to identify the prevalence of NCDs among healthcare workers. One strength of the study is its large sample size (*N* = 3,097), which enhances the internal validity of the findings. The data were collected from patients' EHRs and coded using the ICD-10 and SNOMED systems, reducing the risk of misclassification bias.

### Limitations

Despite its strengths, the study has limitations. As it is a retrospective design, many variables that could act as confounders were missing from the records, such as unhealthy behaviors common among employees, including a sedentary lifestyle, unhealthy food choices, sleep disturbances, smoking, and alcohol use. In addition, the study was conducted within Hamad Medical Corporation, which may limit the generalizability of the findings to other healthcare settings in the country.

## Conclusions

The study reveals a substantial burden of NCDs among healthcare workers in Qatar. The findings showed that older age, female gender, being married, certain nationalities and occupations are associated with a higher likelihood of NCDs. Addressing NCDs among healthcare workers in Qatar requires comprehensive and targeted interventions. Implementing workplace health programs that focus on early detection, lifestyle modifications, and support for mental health may mitigate the burden of NCDs. These efforts are crucial for improving the health and productivity of the workforce, ultimately contributing to the overall economic and social wellbeing of Qatar.

## Data Availability

The raw data supporting the conclusions of this article will be made available by the authors, without undue reservation.
